# Tripterygium wilfordii glycosides ameliorates collagen-induced arthritis and aberrant lipid metabolism in rats

**DOI:** 10.3389/fphar.2022.938849

**Published:** 2022-08-29

**Authors:** Yitian Zhu, Luyun Zhang, Xiafeng Zhang, Dehong Wu, Leiming Chen, Changfeng Hu, Chengping Wen, Jia Zhou

**Affiliations:** ^1^ The Second Clinical Medical College of Zhejiang Chinese Medical University, Hangzhou, China; ^2^ Institute of Basic Research in Clinical Medicine, College of Basic Medical Science, Zhejiang Chinese Medical University, Hangzhou, China; ^3^ The Second Affiliated Hospital of Zhejiang Chinese Medical University, Hangzhou, China; ^4^ Department of Nephrology, Wenzhou Hospital of Integrated Traditional Chinese and Western Medicine, Wenzhou, China

**Keywords:** rheumatoid arthritis, Tripterygium wilfordii glycosides, lipid metabolism, shotgun lipidomics, collagen-induced arthritis

## Abstract

Rheumatoid arthritis (RA) is a chronic inflammatory autoimmune disease, and the dysregulation of lipid metabolism has been found to play an important role in the pathogenesis of RA and is related to the severity and prognosis of patients. Tripterygium wilfordii glycosides (TWG) is extracted from the roots of Tripterygium wilfordii Hook F. with anti-inflammatory and immunosuppressive effects, and numerous clinical trials have supported its efficacy in the treatment of RA. Some evidence suggested that TWG can modulate the formation of lipid mediators in various innate immune cells; however whether it can improve RA-related lipid disorders has not been systematically studied. In the study, type Ⅱ collagen-induced arthritis (CIA) model was used to investigate the efficacy of TWG in the treatment of RA and its effect on lipid metabolism. Paw volume, arthritis score, pathological changes of ankle joint, serum autoantibodies and inflammatory cytokines were detected to assess the therapeutic effect on arthritis in CIA rats. Then, shotgun lipidomics based on multi-dimensional mass spectrometry platform was performed to explore the alterations in serum lipidome caused by TWG. The study showed that TWG could effectively ameliorate arthritis in CIA rats, such as reducing paw volume and arthritis score, alleviating the pathological damages of joint, and preventing the production of anti-CII autoantibodies and IL-1β cytokine. Significant increase in ceramide and decrease in lysophosphatidylcholine were observed in CIA rats, and were highly correlated with arthritis score and IL-1β level. After TWG treatment, these lipid abnormalities can be corrected to a great extent. These data demonstrate that TWG exerts a beneficial therapeutic effect on aberrant lipid metabolism which may provide new insights for further exploring the role and mechanism of TWG in the treatment of RA.

## 1 Introduction

Rheumatoid arthritis (RA) is a chronic autoimmune disease affecting about 0.5–1% of the population worldwide (Symmons et al., 2002; Humphreys et al., 2013), with a high prevalence in women and a considerable disease and social burden including joint pain, disability, high incidence of comorbidities and long-term financial costs (Dougados et al., 2014). The true etiology of RA is complex, involving multiple factors such as pathogen infection, genetics, immunity, *etc.,* and remains to be completely elucidated (McInnes and Schett, 2011; Croia et al., 2019; Jung et al., 2019; Karami et al., 2019). At present, the primary goal of RA treatment is to relieve symptoms and slow down the progress of the disease. A better understanding of disease mechanisms could lead to the development of effective preventive and therapeutic approaches to RA.

Numerous studies have demonstrated that lipids play an important role in the pathogenesis of RA. Some lipid species, like eicosanoids, sphingolipids and lipoxins, etc., are considered to be crucial for the development of arthritic diseases by tightly regulating inflammatory processes (Gerritsen et al., 1998; Serhan et al., 2008; McInnes and Schett, 2011). Peroxidation of membrane phospholipids produces biologically active aldehydes, such as malonaldehyde (MDA) and 4-hydroxynonenal (HNE), which damage the fluidity and permeability of the plasmatic membrane, eventually leading to destruction of cell structure and function (Phaniendra et al., 2015; Quiñonez-Flores et al., 2016). Studies have found that MDA level in RA patients was significantly elevated (Aryaeian et al., 2011; Hassan et al., 2011; Mishra et al., 2012), and positively associated with RA activity (Datta et al., 2014). In addition, alterations in a number of phosphatidylcholine (PC), lysophosphatidylcholine (LysoPC), phosphatidylethanolamine (PE), and sphingomyelin (SM) have been recognized to be correlated with disease activity in RA patients and reflect the therapeutic response to anti-rheumatic drugs (Kosinska et al., 2014; Koh et al., 2022). There are changes in the lipoprotein profiles in RA patients that may lead to increased morbidity and mortality (Toms et al., 2010). About 55–65% of RA patients developed dyslipidemia at an early stage (Curtis et al., 2012; Bag-Ozbek and Giles, 2015; Nowak et al., 2016; Phull et al., 2018), which may be accountable for the higher risk of comorbidities such as cardiovascular disease (CVD) in these patients (Myasoedova et al., 2010; Bag-Ozbek and Giles, 2015; Charles-Schoeman et al., 2015; Łuczaj et al., 2016).

Tripterygium wilfordii Hook F. (TWHF), a traditional herbal medicine, was reported to be effective in the treatment of RA and other immune diseases (Tao and Lipsky, 2000; Jiang et al., 2015). Tripterygium wilfordii glycosides (TWG) is extracted from the roots of TWHF and exhibits anti-inflammatory and immunosuppressive effects (Ma et al., 2007). Research in synovial fibroblasts of arthritis patients suggested that TWG has an effect on decreasing the activity of nuclear factor κ-B (NF-κB), inhibiting gene expression of cyclooxygenase (COX)-2 and inducible nitric oxide synthase (iNOS), reducing the production of prostaglandin E2 (PGE2) and NO, and promoting caspase-3 expression (Yang et al., 2020). The dysregulation of lipid metabolism has been suggested to be involved in the pathogenesis of RA and is related to the severity and prognosis of patients. Improving lipid metabolism can help restore the metabolic homeostasis of RA patients, thereby alleviating the disease and reducing complications. Although TWG has been shown to exert a beneficial therapeutic effect in RA, whether it can improve RA-related lipid disorders has not been studied.

In the present study, type Ⅱ collagen-induced arthritis (CIA) rat model, which has better similarity to human RA due to its chronic disease process (Holmdahl et al., 1992), was used to investigate the efficacy of TWG in the treatment of RA and its effect on lipid metabolism. The changes in paw volume, arthritis score, pathological changes of joint, serum autoantibodies and pro-inflammatory cytokines were detected to assess the efficacy of TWG on collagen-induced arthritis. Multi-dimensional mass spectrometry-based shotgun lipidomics (MDMS-SL) platform was employed to analyze the alterations of serum lipidome induced by TWG. Our results may provide further evidence of the role and mechanism of TWG in the treatment of RA.

## 2 Materials and methods

### 2.1 Reagents

Bovine type Ⅱ collagen (CⅡ) was purchased from Chondrex (Redmond, WA, United States), Freund’s complete adjuvant (FCA) and Freund’s incomplete adjuvant (FIA) were purchased from Sigma (St.Louis, MO, United States). Lipid standards were purchased from Avanti Polar lipids, Inc. (Alabaster, AL, United States). Chromatographic grade methanol and chloroform were purchased from Merck (Darmstadt, Germany). Tripterygium wilfordii glycosides tablets were purchased from Deende Pharmaceutical Co., Ltd. (Zhejiang, China. Lot: 1206101, 10 mg/tablet). The ELISA kit for rat IL-1β was purchased from R&D systems (Minneapolis, MN, United States), and the anti-typeⅡ collagen antibodies (anti-CII) in serum were assayed by ELISA kit (Chondrex, Redmond, WA, United States).

### 2.2 Animal modeling and drug treatment

Female Wistar rats, 7 weeks old, weighing (160 ± 20) g, were provided by the Laboratory Animal Services Center of Zhejiang Chinese Medical University (Hangzhou, China). The animal experiment was approved by the Animal Ethics Committee of Zhejiang Chinese Medical University. After adaptive feeding for 1 week, rats were used to establish collagen-induced arthritis model according to a literature method (Rosloniec et al., 2010). The brief description is as follows: six rats were selected as control group, and the others were primarily immunized with CⅡ emulsified in FCA. About 200 μL of bovine CⅡ emulsion (1.0 mg/ml) was injected intradermally at the base of the tail. 1 week after the primary immunization, a booster immunization was given with 150 μL of CⅡ emulsion in FIA (1.0 mg/ml). The control group was injected with an equal volume of normal saline.

**FIGURE 1:**
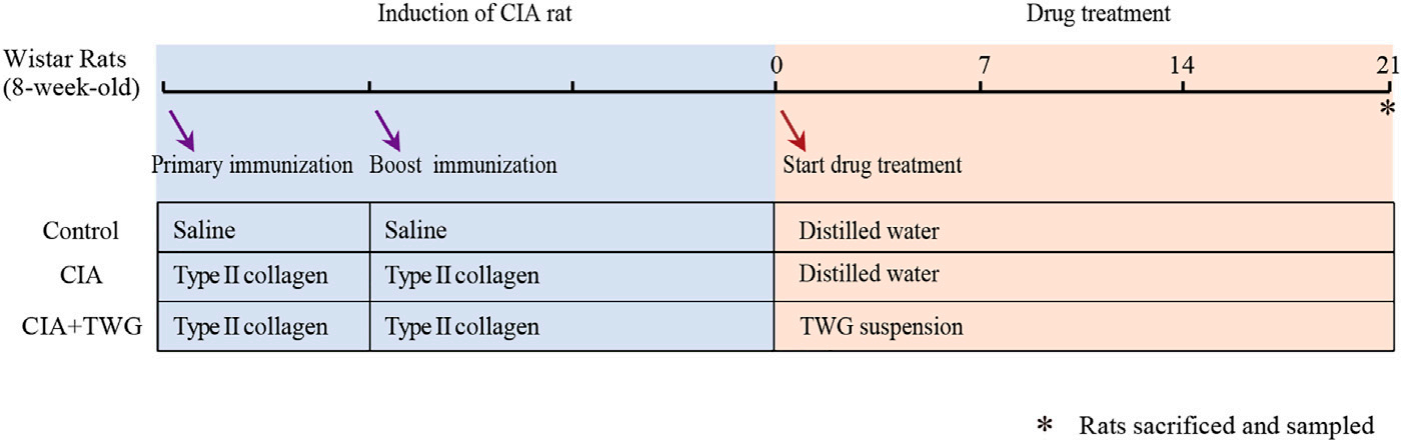
Experimental flow chart for evaluating the effect of TWG on collagen-induced arthritis in Wistar rats.

21 days after primary immunization, the rats with induced arthritis (arthritis score>6) were randomly divided into model group (CIA group, *n* = 6) and CIA + TWG group (*n* = 6). The CIA + TWG group was administrated with 6 mg/kg of TWG per day, equivalent to regular human dose of 1 mg/kg per day. The TWG suspension was prepared from tablet powder dissolved in distilled water. The CIA group and control group were given orally the same volume of distilled water. The rats were anesthetized with chloral hydrate (10%, w/v) and blood was collected from abdominal aorta after 21 days of intervention. The serum was separated by centrifugation for 10 min at 1,200 g and all samples were stored in refrigerator at -80°C. The detailed experimental process and grouping information are shown in Figure 1.

### 2.3 Evaluation of arthritis severity

After primary immunization, arthritis scores were measured every 7 days. Arthritis severity of each limb was graded on a 0–4 scale according to the modified method of (Wang et al., 2021): no swelling (0 points); mild swelling of the little toe joints (1 point); swelling of the toe joints and foot plantar (2 points); swelling of the foot below the ankle (3 points); swelling of entire foot, including the ankle (4 points). The arthritis score of the rat was obtained by summing the scores of the four limbs, and a score equal to or greater than 6 points is considered to be successful modeling, with a maximum score of 16 points (4 × 4). In addition, at 0, 7, 14 and 21 days after drug treatment, the paw volume of the right hind limb was measured with a toe volume meter as the paw swelling index.

At the end of the animal experiment, ankle joints of rats were taken out. X-ray images (CARESTREAM Image Station System, Carestream Health, Inc., United States) were taken to observe the morphological changes of the joints. Furthermore, ankle joints were flushed with PBS, and fixed by 4% paraformaldehyde for 48 h. After decalcification, paraffin sections were made and stained with hematoxylin and eosin (H&E) to evaluate the histological changes of ankle joints.

### 2.4 Detection of IL-1β and anti-CII antibodies levels

An aliquot (100 µL) of serum samples from different groups or rats were collected to measure the levels of IL-1β and anti-CII antibodies by ELISA kits according to the manufacturers’ instructions.

### 2.5 Lipid extraction, analysis and data preprocessing

Serum lipids were extracted by the modified Bligh-Dye protocol (Bligh and Dyer, 1959) in the presence of internal standards as described in the reference (Yang et al., 2009). The chloroform phase containing lipids was collected. The extraction process was repeated twice, and the lipid extracts were combined and evaporated under a nitrogen stream. The dried lipid extracts were redissolved in 2 μL of chloroform/methanol (1:1, v/v), sealed with nitrogen, and stored at -20°C until analysis.

The analysis of serum lipids was carried out on a triple-quadrupole mass spectrometer (TSQ Quantiva, Thermo Scientific) connected to an automated nanospray ion source (NanoMate, Advion Bioscience) according to the reference (Han et al., 2005). Before lipid analysis, each lipid extract was further diluted with chloroform/methanol/isopropyl alcohol (1:2:4, *v/v/v*). Various species of lipids were characterized and quantified by MDMS-SL according to the reference (Yang, et al., 2009).

All the mass data were acquired through different sequence subroutines running by Xcalibur software. Data preprocessing, including baseline calibration, de-isotope peak, peak intensity calculation, etc., was carried out according to the published reference (Han, et al., 2005).

### 2.6 Statistical analysis

Principal component analysis (PCA) based on the phospholipid profiles was carried out by SIMCA-P 14.1 (Umetrics AB, Umea, Sweden) to generally observe the distribution of samples from the control, CIA and CIA + TWG groups after mean centering. Furthermore, orthogonal partial least squares discriminant analysis (OPLS-DA) was employed to distinguish groups and screen the discriminant serum lipids. Permutation test was used to verify whether the model was over-fitted. Lipids with a variable importance in projection (VIP) value of the OPLS-DA model greater than 1.0 were considered to play an important role in the classification of different groups. Both *p* values and VIP values were taken as criteria for screening potential differential lipids, and VIP >1.0 and *p* < 0.05 were used as cutoff.

To investigate the statistical significance in the levels of the paw volume, arthritis scores, IL-1β, anti-CII and lipids between control, CIA and CIA + TWG groups, ANOVA followed by a Bonferroni post hoc test for pairwise comparisons were performed using SPSS 18.0 (International Business Machines Corp., Armonk, United States). And *p* < 0.05 was considered statistically significant. In addition, Pearson’s correlation between the arthritis score, IL-1β, anti-CII and differential lipids were analyzed.

### 2.7 Role of the funding source

No funding source had any role in study design; in the collection, analysis, and interpretation of data; in the writing of the report; and in the decision to submit the paper for publication. The corresponding author had full access to all the data in the study and had final responsibility for the decision to submit for publication.

## 3 Results

### 3.1 Exploring the change of paw volume, arthritis score, IL-1β and anti-CII after Tripterygium wilfordii glycosides treatment

**FIGURE 2:**
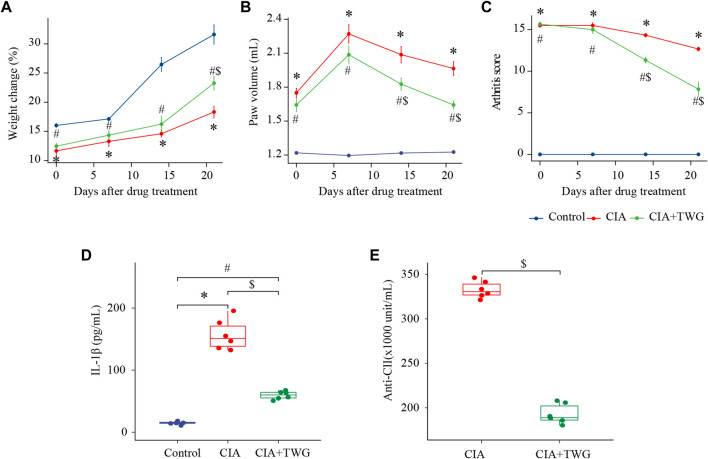
Comparison of percentage of weight change **(A)**, paw volume **(B)**, arthritis score **(C)**, IL-1β level **(D)**, anti-CⅡ level **(E)** in rats of the control, CIA and CIA + TWG groups. The percent change in weight was calculated as (weight after drug treatment-weight before immunization)/weight before immunization. ∗ represents *p* < 0.05 between the control and CIA groups, # represents *p* < 0.05 between the control and CIA + TWG groups, and $ represents *p* < 0.05 between the CIA and CIA + TWG groups.

During the experiment, we continued to observe the general condition of the rats. The rats in the control group were in good condition, with shiny fur and free movement. 5–7 days after the booster immunization with collagen, the CIA rats gradually developed polyarthritis, and showed fatigue, weight loss, swelling of foot joints, lameness and mobility impairment. After 21 days of treatment, the CIA + TWG group had smoother fur, significantly increased body weight (*p* < 0.05, Figure 2A), and improved mobility compared with the CIA group.

The severity of arthritis was measured by the paw volume and arthritis score, with greater paw volume and higher arthritis score indicating more severe disease. After modeling, the paw volume of the right hind foot and arthritis score in the CIA group were significantly higher than those in the control group (*p* < 0.01, *p* < 0.01); after 14 days of treatment, compared with the CIA group, the paw volume and arthritis score of CIA + TWG group began to decrease significantly (*p* < 0.05, *p* < 0.01); after 21 days of treatment, the paw volume and arthritis score in the CIA + TWG group continued to decrease (*p* < 0.01, *p* < 0.01), suggesting that the joint swelling was alleviated by TWG (Figures 2B,C).

Then, we investigated the change of pro-inflammatory cytokine in CIA rats. In the study, serum level of IL-1β in CIA group was significantly increased; 21 days after TWG treatment, the IL-1β level was remarkably decreased as compared with the CIA group (*p* < 0.01, Figure 2D), indicating that TWG could inhibit the production of IL-1β to suppress the inflammatory response.

In addition, we examined the levels of anti-CII antibodies in the CIA and CIA + TWG groups after 21 days of treatment. Compared with the CIA group, the level of anti-CⅡ antibodies was relatively lower after the treatment of TWG (*p* < 0.01, Figure 2E), indicating that TWG could prevent the antibody response mediated by collagen.

**FIGURE 3:**
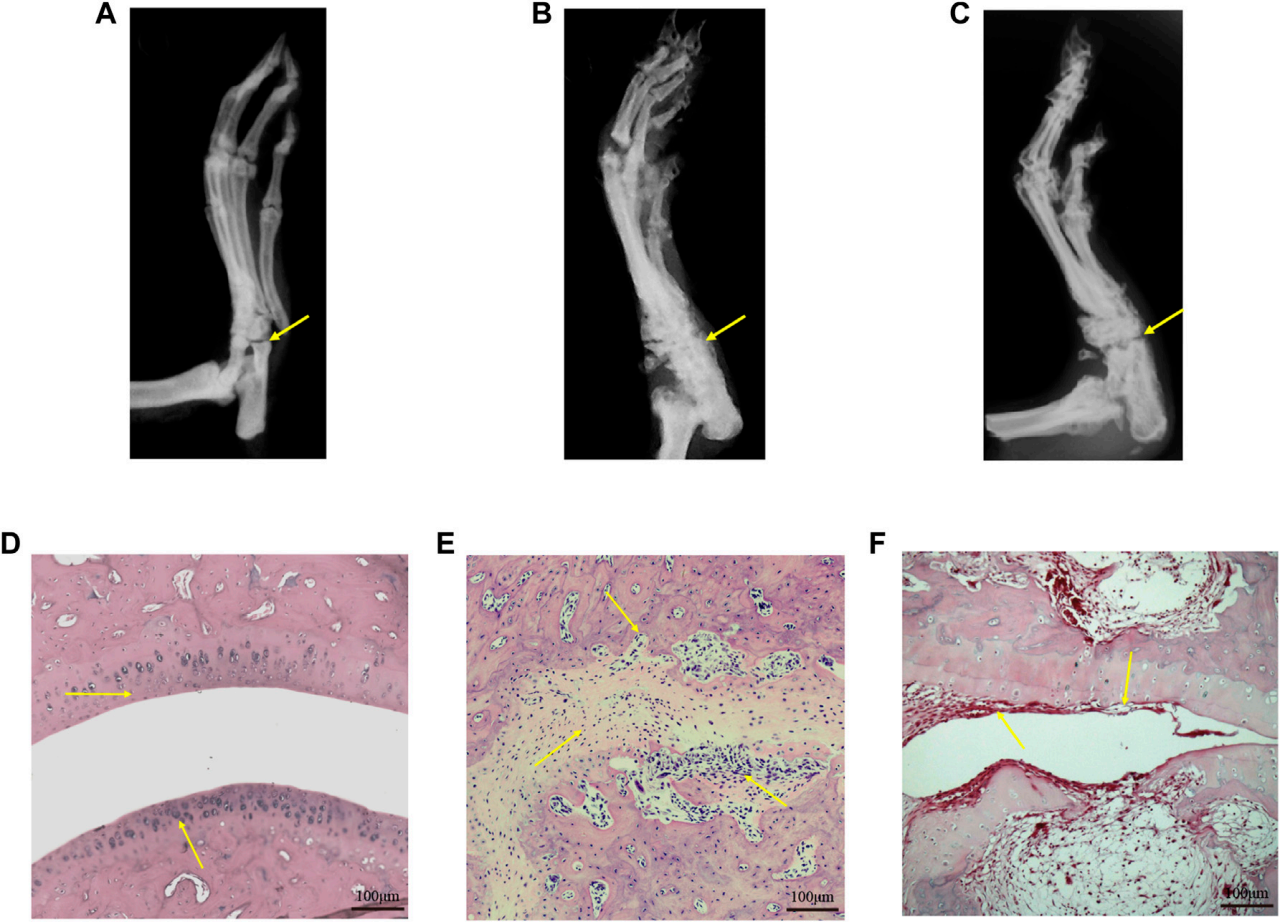
Representative X-ray images and H&E stained histological images of ankle joints in rats of the control [**(A)** and **(D)**], CIA [**(B)** and **(E)**] and CIA + TWG [**(C)** and **(F)**] groups.

X-ray images showed that the CIA group developed severe joint swelling, joint deformity, joint space narrowing and other arthritis-related joint characteristics; after 21 days of treatment, the CIA + TWG group had mild joint space narrowing, joint swelling, joint deformity relief, etc., (Figures 3A–C)*.* The H&E stained histological of ankle joint showed that there were narrowed joint space, significant proliferation of fibrous connective tissue and infiltration of inflammatory cells, and severe erosion of articular cartilage and bone in the CIA group. TWG could alleviate the pathological damages of joint tissues, including reducing inflammatory cell infiltration, and relieving articular cartilage and bone injury (Figures 3D,E).

### 3.2 Alterations of total amounts of each lipid species after Tripterygium wilfordii glycosides treatment

**FIGURE 4:**
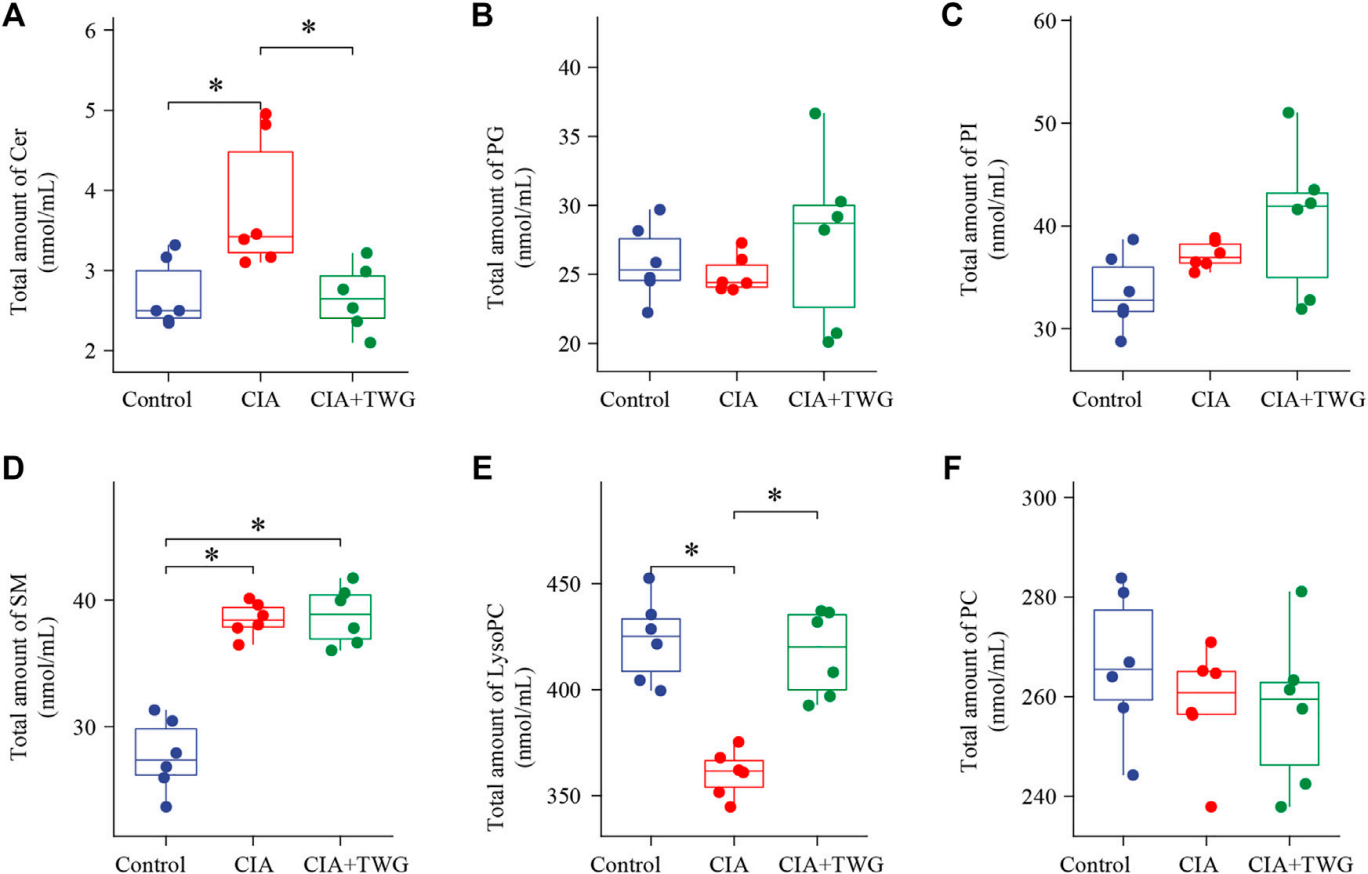
Comparison of the total amounts of individual Q18 lipid species in rats of the control, CIA and CIA + TWG groups. **(A)** Cer, **(B)** PG, **(C)** PI, **(D)** SM, **(E)** LysoPC, **(F)** PC. ∗*p* < 0.05.

Lipids in rat serum were analyzed by MDMS-SL. After data preprocessing such as baseline correction and peak intensity calculation, nearly 100 kinds of lipid molecules belonging to 6 lipid species with high response in mass spectrometry were quantified, including SM, PC, LysoPC, ceramide (Cer), phosphatidylinositol (PI) and phosphatidylglycerol (PG). The total amounts of each lipid species in different groups were calculated respectively. It was found that the most abundant phospholipids are PC and LysoPC among the 6 phospholipid species (Figure 4). As compared to the control group, the total SM level and Cer level was significantly up-regulated in the CIA group (*p* < 0.05), while the total LysoPC level was significantly down-regulated (*p* < 0.05). 21 days after TWG treatment, the total LysoPC level was increased to that of the control group. In addition, TWG treatment reduced the total level of Cer (Figure 4).

### 3.3 Visualization of the difference of lipid profile after Tripterygium wilfordii glycosides treatment

**FIGURE 5:**
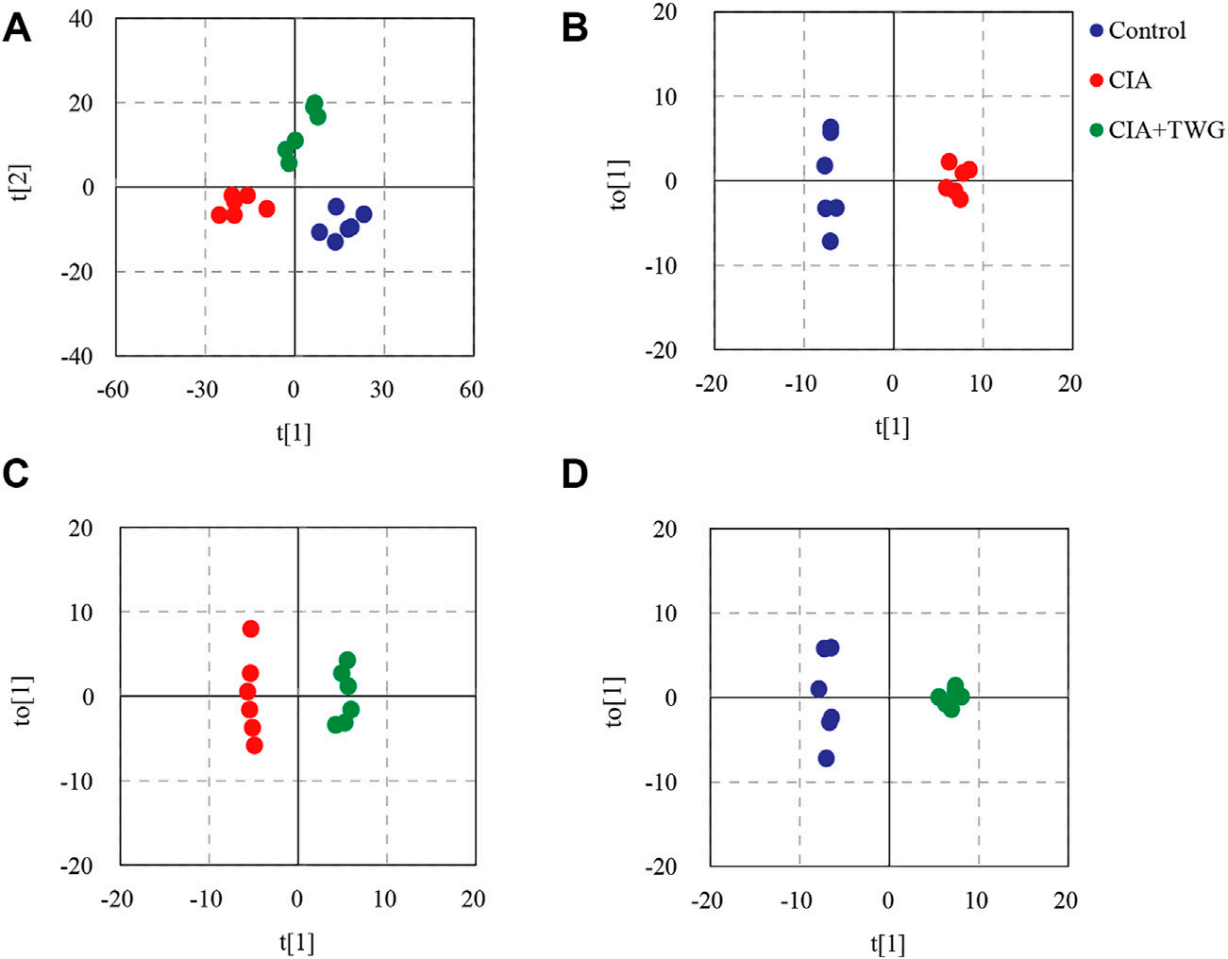
PCA and OPLS-DA based on the serum lipid profiles. **(A)** PCA score plot of the control, CIA and CIA + TWG groups, **(B)** OPLS-DA score plot of the control and CIA groups, **(C)** OPLS-DA score plot of the CIA and CIA + TWG groups, **(D)** OPLS-DA score plot of the control and CIA + TWG groups.

In order to display the overall distribution and clustering of the samples from the control group, the CIA group and CIA + TWG group, the lipid data were subjected to PCA after mean centering. Principal components 1 and 2 explained 53.7% and 25.5% of the variance, respectively. The PCA score plot is shown in Figure 5A, it was observed that there is a clear separation in the lipid profiles between the CIA group and the control group, reflecting the significant changes in serum lipid metabolism in rats after the injection of collagen. The lipid profile of CIA + TWG group tends to be closer to that of the control group, but it is still different from that of the control group.

### 3.4 Alterations of serum lipids associated with collagen immunization and/or Tripterygium wilfordii glycosides treatment

To reveal the lipid alterations caused by collagen immunization and TWG treatment, OPLS-DA was performed between the control group, CIA group and CIA + TWG groups. Permutation tests indicated good fitness of the OPLS-DA models in revealing the alterations in serum lipids. OPLS-DA score plots showed both collagen induction and TWG treatment led to changes in lipid profile (Figures 5B–D). Differential lipids were screened out based on the VIP values of OPLS-DA models (VIP>1) and *p* values of significance tests (*p* < 0.05).

**FIGURE 6:**
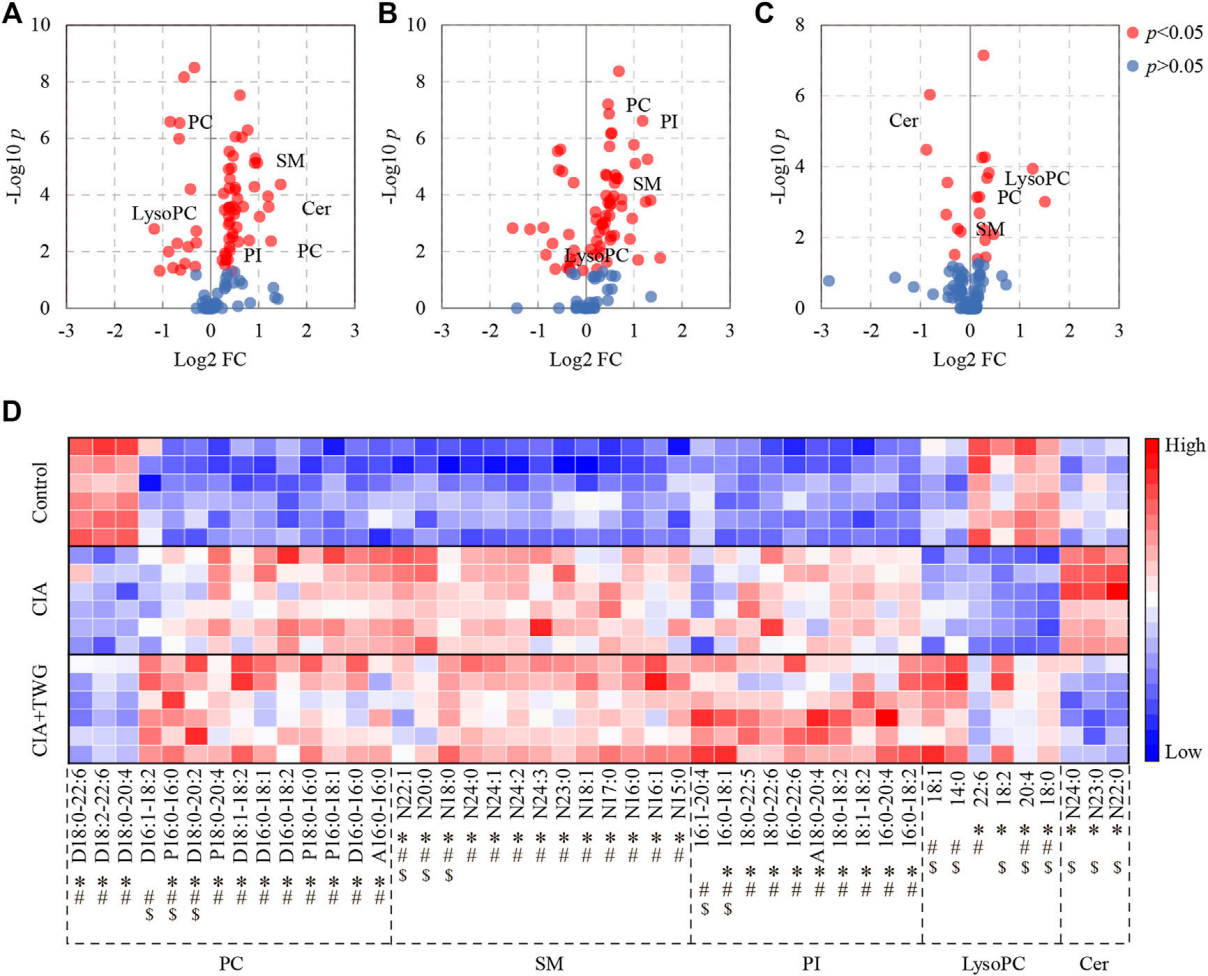
Significantly altered serum lipids related to collagen immunization and TWG treatment. Volcano plot showing the lipids with significant differences between the control and CIA groups **(A)**, between the control and CIA + TWG groups **(B)**, between the CIA and CIA + TWG groups **(C)**, and heatmap showing the expression pattern of each differential lipid **(D)**. Red color indicates high concentration of lipid and blue color indicates low concentration. ∗ represents *p* < 0.05 between the control and CIA groups, # represents *p* < 0.05 between the control and CIA + TWG groups, and $ represents *p* < 0.05 between the CIA and CIA + TWG groups.

The collagen-induced abnormal lipids were selected by comparing the CIA group with the control group. Finally, 42 kinds of altered lipids were identified. There were significant decreases in 4 kinds of LysoPC (18:0, 20:4, 18:2 and 22:6) and 3 kinds of PC (D18:0–20:4, D18:2–22:6 and D18:0–22:6), increases in 3 kinds of Cer (N22:0, N23:0 and N24:0), 9 kinds of PI (16:0–18:2, 16:0–20:4, 18:1–18:2, 18:0–18:2, A18:0–20:4, 16:0–22:6, 18:0–22:6, 18:0–22:5 and 16:0–18:1), 13 kinds of SM (N15:0, N16:1, N16:0, N17:0, N18:1, N23:0, N24:3, N24:2, N24:1, N24:0, N18:0, N20:0 and N22:1) and 10 kinds of PC (A16:0–16:0, D16:0–16:0, P16:0–18:1, P18:0–16:0, D16:0–18:2, D16:0–18:1, D18:1–18:2, P18:0–20:4, D18:0–20:2 and P16:0–16:0) in CIA rats (Figure 6).

The serum lipids related to TWG treatment were screened out by comparing the CIA group with the CIA + TWG group. It was found that 16 kinds of lipids were altered after the treatment of TWG. Among them, 5 kinds of sphingolipids were reduced after treatment, including 3 kinds of Cer (N22:0, N23:0 and N24:0) and 2 kinds of SM (N20:0 and N22:1); 11 kinds of glycerophospholipids were elevated, such as LysoPC (18:0, 20:4, 18:2, 14:0 and 18:1), PI (16:0–18:1 and 16:1–20:4), PC (D18:0–20:2, P16:0–16:0 and D16:1–18:2) and SM (N18:0) (Figure 6).

### 3.5 Effects of Tripterygium wilfordii glycosides treatment on the collagen-induced abnormal lipids

The effects of TWG on the collagen-induced abnormal lipids were also investigated (Figure 6). TWG treatment adjusted the levels of 8 kinds of lipids closer to the normal level, including down-regulation of N22:0 Cer, N23:0 Cer, N24:0 Cer, N20:0 SM and N22:1 SM, up-regulation of 18:0 LysoPC, 20:4 LysoPC and 18:2 LysoPC; however, it caused the levels of 16:0–18:1 PI, N18:0 SM, D18:0–20:2 PC and P16:0–16:0 PC to be further away from the normal level. These results indicated that the abnormal lipid metabolism in CIA rats could be alleviated by TWG to a certain extent, especially Cer and LysoPC species.

### 3.6 Significant associations of arthritis score, IL-1β, anti-CII antibodies with the levels of those differential lipids

**FIGURE 7:**
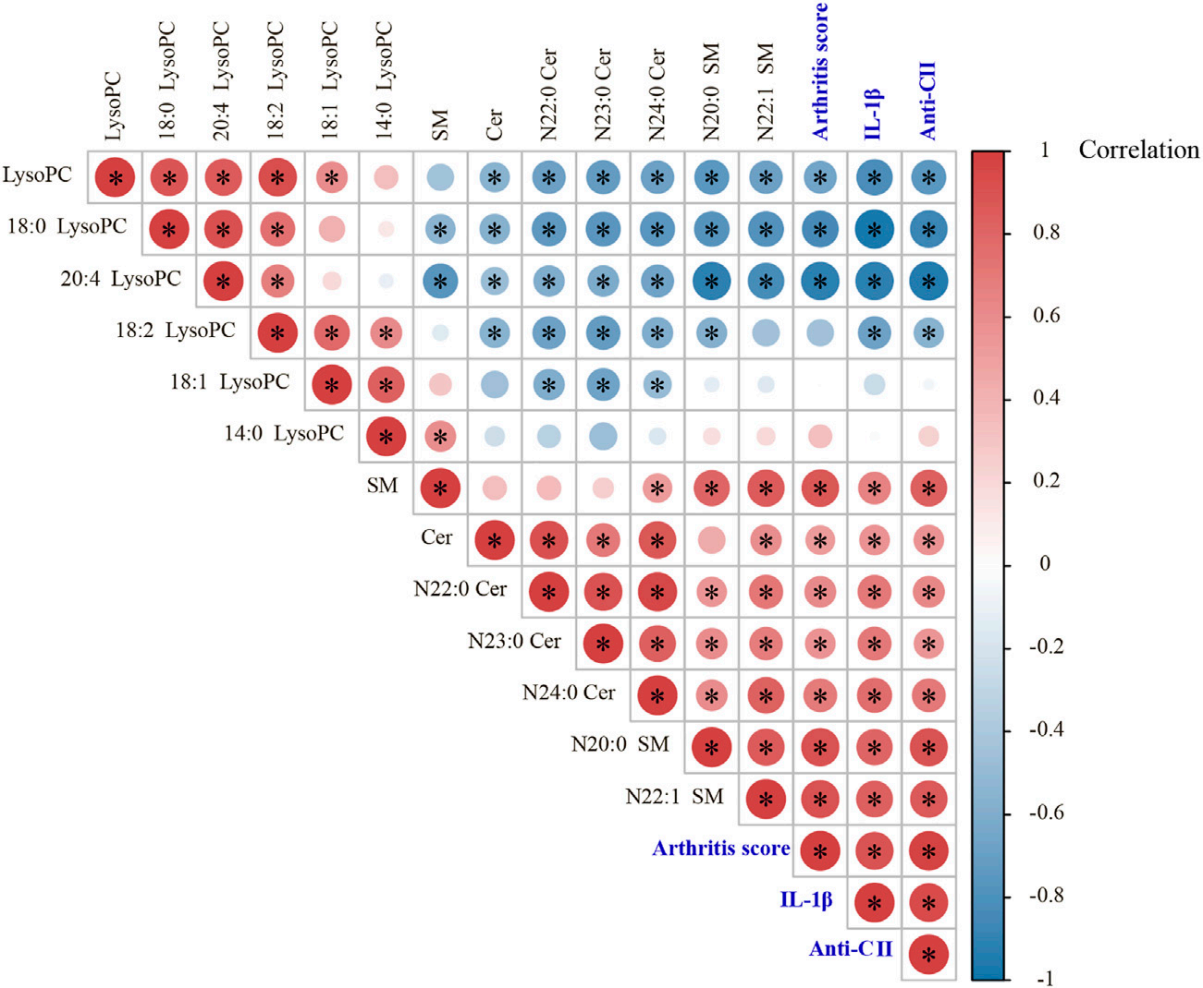
Pearson correlation analyses between arthritis score, IL-1β, anti-CII antibodies and those significantly changed lipids in different groups of rats.

In the study, we found there was a significant positive correlation between IL-1β and arthritis score, which can reflect the severity of joint swelling. Among those screened lipids, the levels of Cer (N24:0, N23:0 and N22:0), SM (N22:1 and N20:0) were positively correlated with IL-1β level and arthritis score, as were the total amounts of SM species and Cer species; while LysoPC (20:4 and 18:0) and the total amount of LysoPC species were negatively correlated with IL-1β and arthritis score. After collagen immunization, antibody response against CII was induced, and TWG treatment downregulated the level of anti-CII antibodies. There is an association between LysoPC, SM, Cer and anti-CII antibodies existed in CIA rats (*p* < 0.05, Figure 7).

## 4 Discussion

CIA is an extensively used animal model of autoimmune arthritis. The pathological manifestations of CIA models are progressive synovitis and synovial hyperplasia, inflammatory cell infiltration, cartilage destruction, and finally lead to joint injury and stiffness; these characteristics are more similar to those of clinical RA (Holmdahl, et al., 1992). CIA model is established by immunizing genetically susceptible strains of mice/rats with CII. CII activates innate and adaptive immune responses, which have a primary role in the initiation and pathogenesis of RA in CIA model. Some studies found that anti-CII antibodies are present in the serum and synovial fluid of RA patients precede the onset of joint symptoms (Mullazehi et al., 2007; Whittingham et al., 2017). Patients with positive anti-CII antibodies exhibited higher disease activity and more severe symptoms (Mullazehi, et al., 2007). In the study, CIA rat model was used to investigate the efficacy of TWG. Anti-CII antibodies can be detected in CIA rats which implied that the immune response is induced by CII immunization. IL-1β is the initiating factor of inflammation and regulates a variety of cytokines, cell adhesion molecules and inflammatory mediators. Previous studies have shown that the level of IL-1 in the circulation of RA patients is higher than that of other chronic inflammatory joint diseases (Kay and Calabrese, 2004), and is associated with bone erosion and cartilage destruction in RA (Guo et al., 2018). Our results demonstrated that TWG could alleviate the severity of the disease, including reducing joint swelling, repairing joint injury, decreasing the generation of serum autoantibodies (anti-CII) and the secretion of pro-inflammatory cytokines (IL-1β).

As energy sources, structural constituents and signaling molecules, lipids participate in the regulation of many important biological processes, such as cell growth, proliferation, differentiation, death, etc., (Wymann and Schneiter, 2008; Han, 2016). The disorders of lipid metabolism may lead to abnormalities in signaling, inflammation and autoimmune responses (Wymann and Schneiter, 2008). In this study, shotgun lipidomics revealed that the total amounts of Cer and SM species were increased in CIA rats, and were positively correlated with pro-inflammatory cytokine IL-1β. SM is an important component of cell biofilms and plasma lipoproteins, and plays a pro-inflammatory role by enhancing the expression of COX-2 and encoding genes related to inflammatory cytokines (Miltenberger-Miltenyi et al., 2020). SM can be hydrolyzed into Cer, which is involved in TNFα-mediated activation of NF-κB and RANKL-mediated osteoclast differentiation to promote the development of RA (Qu et al., 2018). Recent studies have found that the levels of Cer and SM species in synovial fluid of patients with RA and osteoarthritis were increased as compared to the healthy controls, which is consistent with the role of Cer and SM in inflammation (Aletaha et al., 2010). The study showed that the treatment of TWG could totally reverse the elevation of Cer level, and greatly reduce the levels of some SM (N20:0 and N22:1) molecules, which may facilitate the amelioration of inflammation and joint swelling in CIA rats. Ceramidase is essential for converting Cer to sphingosine, and some evidence suggested that TWG can interact with ceramidase to regulate the level of Cer (Qian et al., 2022).

LysoPC is generated by phospholipase A2 (PLA2)-catalyzed degradation of membrane PC (Law et al., 2019), and plays a chemotactic role at the inflammatory site, thus boosting inflammatory response. However, it was reported that low level of LysoPC was observed in active RA patients, which might be related to the decrease of PLA2 activity (Lourida et al., 2007; Koh, et al., 2022). Our study also showed that the total amount of LysoPC was significantly decreased in CIA rats, and accordingly, the levels of most PC molecules were elevated to some extent. LysoPC is a major component of oxidized low density lipoprotein (oxLDL), which has been proposed as a critical pathogenic factor of atherosclerosis (Law, et al., 2019). Evidence suggested that there was an inverse correlation between LysoPC and the risk of CVD (Lee et al., 2013; Stegemann et al., 2014), which has a high incidence in RA patients (Aviña-Zubieta et al., 2008). Intriguingly, the reduced level of LysoPC in CIA rats can be corrected after the treatment of TWG. Network pharmacology research has found that some absorbed components of TWG, such as hypoglaulide, triptotriterpenic acid A, Wilforlide A, can target PLA2G10, PLA2G2A and PLA2G1B, thereby interfering with glycerol phospholipid metabolism and ether lipid metabolism, which in turn led to changes in the level of lysoPC and PC (Qian, et al., 2022). Taken together, the observed lipid profiles suggest an ameliorative effect of TWG on lipid disorders associated with RA, but do not provide a mechanistic explanation for the finding. Whether this is a cause or a consequence of joint inflammation remains to be investigated further, which is one of the limitations of this study.

## 5 Conclusion

The present study showed that TWG could effectively relieve the joint swelling, repair joint injury, and prevent the production of anti-CII autoantibodies and the secretion of IL-1β cytokine in CIA rats. Moreover, TWG could improve aberrant lipid metabolism caused by collagen immunization, including down-regulating Cer level and up-regulating LysoPC level. These results suggest that TWG exerts a beneficial therapeutic effect on lipid metabolism disorders, and further research is needed to better explain the biological mechanisms underlying these findings.

## Data Availability

The original contributions presented in the study are included in the article/supplementary material, further inquiries can be directed to the corresponding authors.
